# Supplementary material for: An RPA-CRISPR/Cas12a-based rapid and sensitive nucleic acid method for detection of Toxoplasma gondii in tissue and blood samples
Source: Microbiol Spectr. 2025 Nov 28;14(1):e01550-25. doi: 10.1128/spectrum.01550-25 (PMC12772379; doi:10.1128/spectrum.01550-25)
Supplement: Supplemental material — Supplemental figures and tables. [file spectrum.01550-25-s0001.docx]

Supplemental Materials

**RPA-CRISPR/Cas12a-based rapid and sensitive nucleic acid detection method of** ***Toxoplasma gondii* in tissue and blood samples**

Yilin Wang^1,2,3^, Ziyang Qin^1,2,3^, Qinglin Wang^1,2,3^, Chunhao Gu^1,2,3^, Fuchang Yu^1,2,3,4^, Yayun Wu^1,2,3^, Long xian Zhang^1,2,3,*^

*^1^ College of Veterinary Medicine, Henan Agricultural University, Zhengzhou, Henan, P. R. China*

*^2^ International Joint Research Laboratory for Zoonotic Diseases of Henan, Zhengzhou, P. R. China*

*^3^ Key Laboratory of Quality and Safety Control of Poultry Products, Ministry of Agriculture and Rural Affairs, Zhengzhou, Henan, P. R. China*

*^4^ College of Animal Science, Tarim University, Alar, Xinjiang, P. R. China*

* Corresponding author

College of Veterinary Medicine, Longzihu Campus of Henan Agricultural University, No. 15 Longzihu University Area, Zhengzhou New District, Zhengzhou, 450046, P. R. China.

Tel.: 86-371-56990163; Fax: 86-371-56990163;

E-mail: zhanglx8999@henau.edu.cn

**TABLE S1** Information on the tissue and blood samples used in this study

| Number of samples | Types of samples | Host | Sample source |
| --- | --- | --- | --- |
| 1 | Testicle | Kunming mice | The Pathology Laboratory of Henan Agricultural University |
| 2 | Testicle | Kunming mice |  |
| 3 | Liver | binary hybrid pig | The Poultry Disease Research Institute of Henan Agricultural University |
| 4 | Spleen |  |  |
| 5 | Kidney |  |  |
| 6 | Kidney | binary hybrid pig | Xuchang, Henan |
| 7 | Lung |  |  |
| 8 | Heart |  |  |
| 9 | Brain |  |  |
| 10 | Thyroid gland |  |  |
| 11 | Spleen | Kunming mice | Stored in our laboratory |
| 12 | Intestinal lymph nodes |  |  |
| 13 | Spleen | Kunming mice | Stored in our laboratory |
| 14 | Intestinal lymph nodes |  |  |
| 15 | Blood | Hu sheep | A sheep farm in Huzhou, Zhejiang |
| 16 | Blood | Hu sheep |  |
| 17 | Blood | Hu sheep |  |
| 18 | Blood | Kunming mice | Stored in our laboratory |
| 19 | Blood | Kunming mice |  |
| 20 | Blood | Kunming mice |  |

**TABLE S2** Nucleotide sequences used in this study

| Name | | | Sequence (5’-3’) |
| --- | --- | --- | --- |
| Target sequence | | | TTCAAGGGAAGAGATCCAGCAGATCTC |
| RPA primers | | |  |
| 1 | F104-133 | | GAAGTATTTGAGGTCATATCGTCCCATGAA |
|  | R304-335 | | AATACAGCTCTTGTTATGATGCAACACGTACC |
| 2 | F101-132 | | AGAGAAGTATTTGAGGTCATATCGTCCCATGA |
|  | R297-328 | | CTCTTGTTATGATGCAACACGTACCCTTCTTC |
| 3 | F105-134 | | AAGTATTTGAGGTCATATCGTCCCATGAAG |
|  | R357-386 | | ATACTAGGTGGCTTTTCTGGAGGTACATTT |
| 4 | F91-120 | | CCTTACTGCAAGAGAAGTATTTGAGGTCAT |
|  | R354-385 | | TACTAGGTGGCTTTTCTGGAGGTACATTTCAC |
| 5 | F102-132* | | GAGAAGTATTTGAGGTCATATCGTCCCATGA |
|  | R295-326* | | CTTGTTATGATGCAACACGTACCCTTCTTCTG |
| PCR primers | | |  |
| B1 | | B1-Tg1 | TGTTCTGTCCTATCGCAACG |
|  |  | B1-Tg2 | ACGGATGCAGTTCCTTTCTG |
|  |  | B1-Tg3 | TCTTCCCAGACGTGGATTTC |
|  |  | B1-Tg4 | CTCGACAATACGCTGCTTGA |
| crRNA | | |  |
| crRNA-Tg | | | AAUUUCUACUGUUGUAGAUAAGGGAAGAGAUCCAGCAGAUCUC |
| crRNA-Tg-F | | | gaaatTAATACGACTCACTATAgggAATTTCTACTGTTGTAGATAAGGGAAGAGATCCAGCAGATCTC |
| crRNA-Tg-R | | | GAGATCTGCTGGATCTCTTCCCTTATCTACAACAGTAGAAATTcccTATAGTGAGTCGTATTAatttc |

Underline indicates the PAM region.

* Corresponds to the optimum RPA primers used in this study


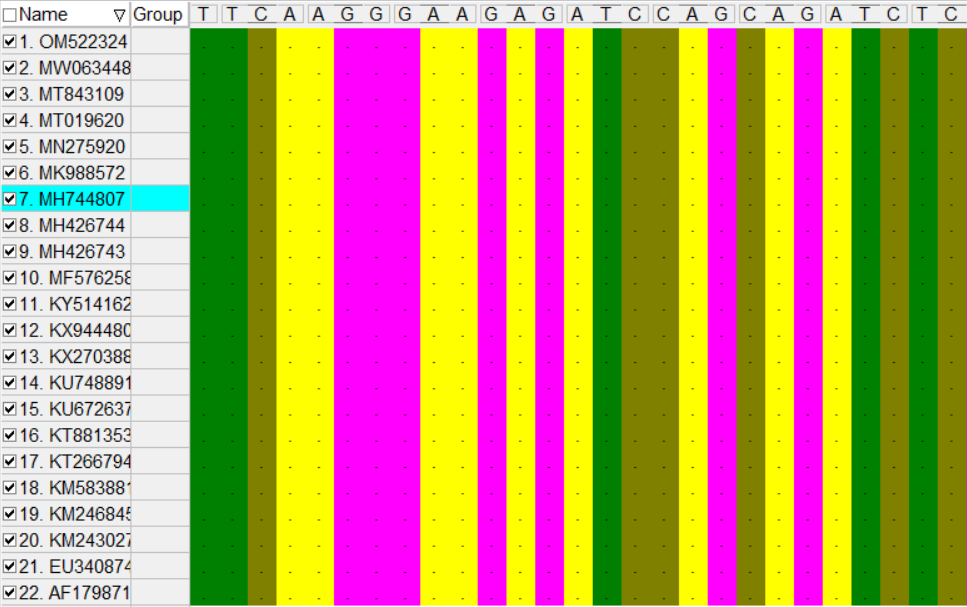


**FIG S1** Design of the specific crRNA probe based on the B1 gene sequences of *T. gondii*.


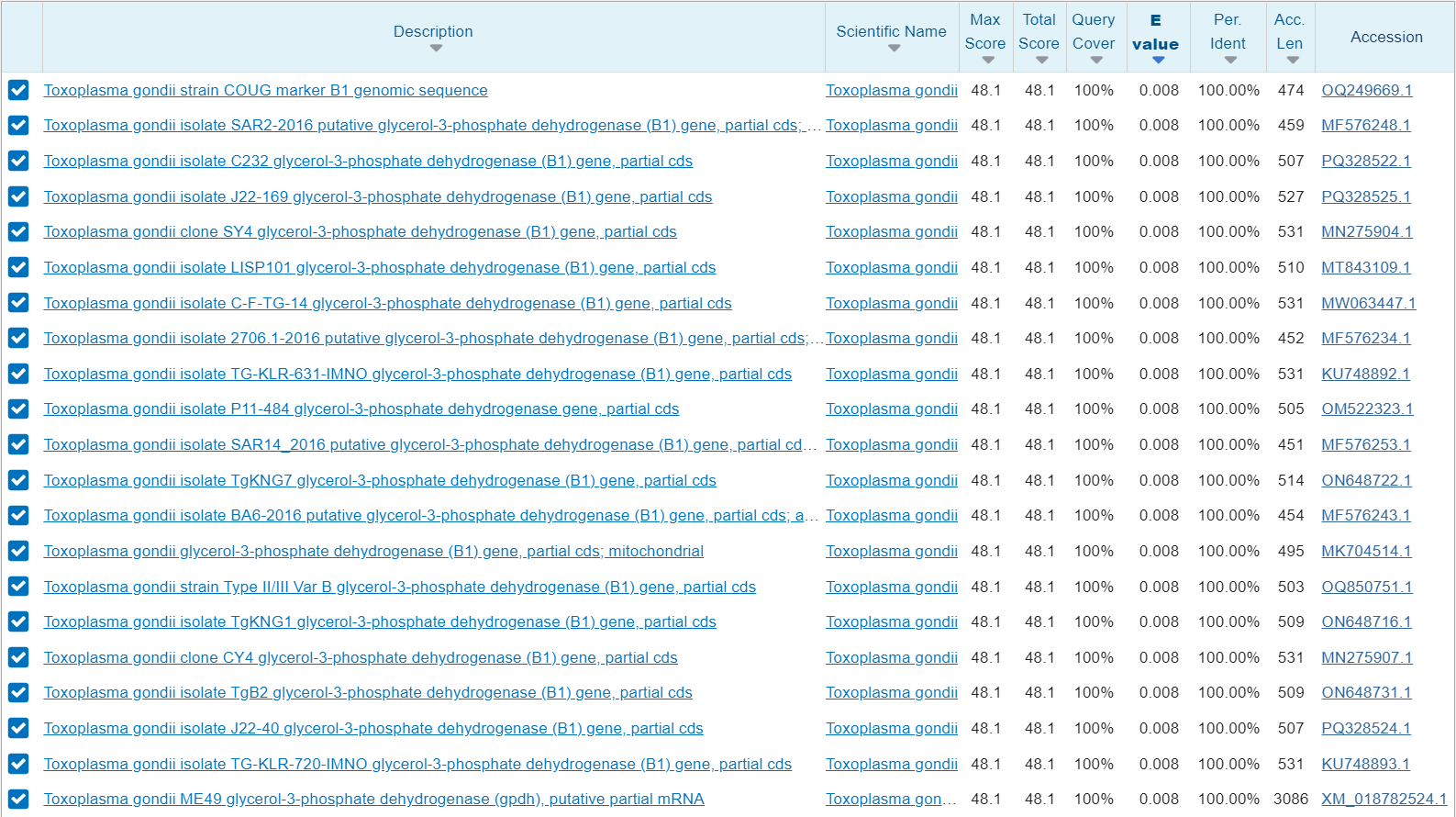


**FIG S2** The BLAST analysis partial result of the crRNA sequence.


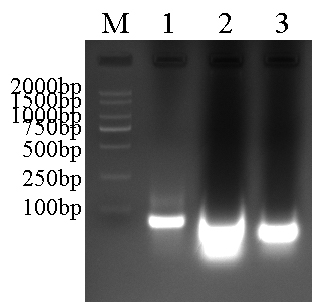


**FIG S3** The agarose gel electrophoresis results of annealing product, transcription product, and purified product. M: DL2000 DNA Marker; 1: annealing product; 2: transcription product; 3: purified product.


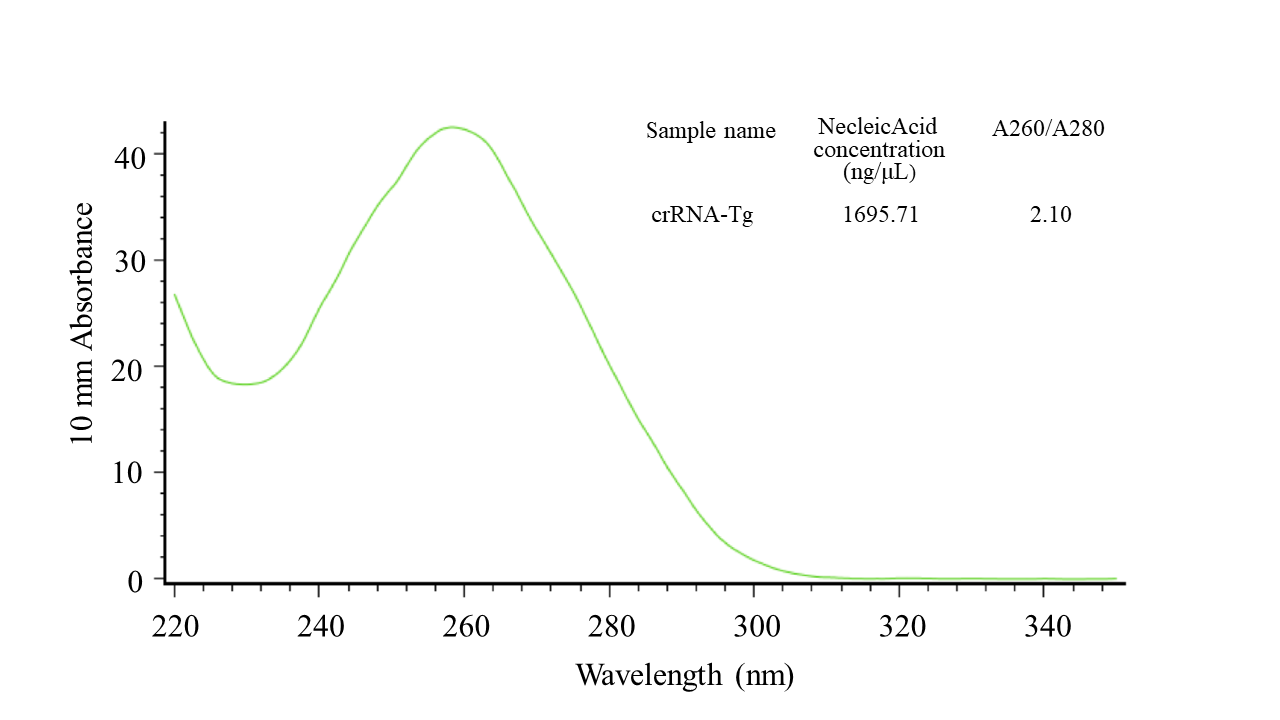


**FIG S4** Absorbance curves of purified crRNA. crRNA was transcribed from crDNA annealed from two reverse complementary single-strand oligonucleotides. The transcribed crRNA was treated with DNase I and was purified using the NucAway™ Spin Column.


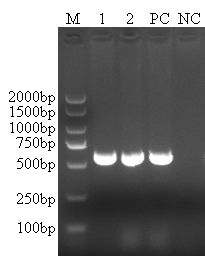


**FIG S5** The results of PCR amplification of *T. gondii* based on the B1 locus. M: DL2000 DNA Marker; 1-2: DNA extracted from tachyzoites; PC: positive control; NC: negative control.


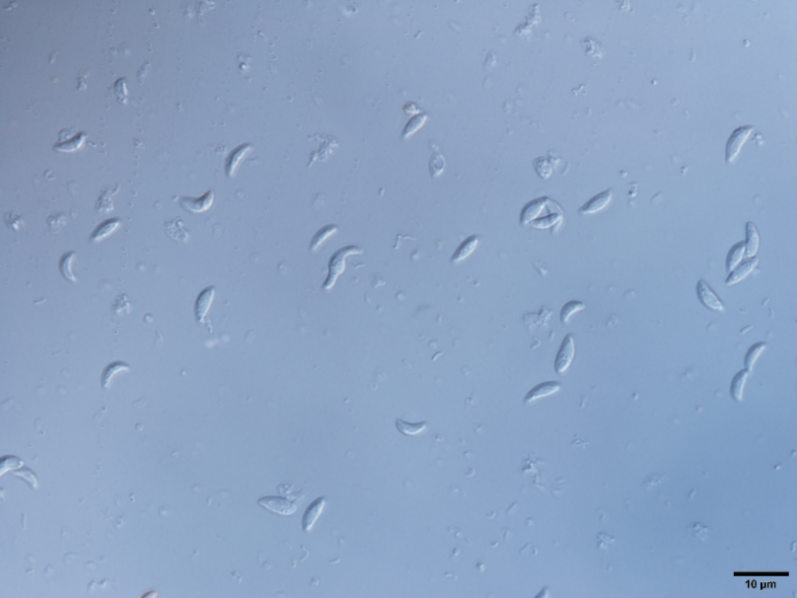


**FIG S6** Purified tachyzoite of *T. gondii* (1000×)


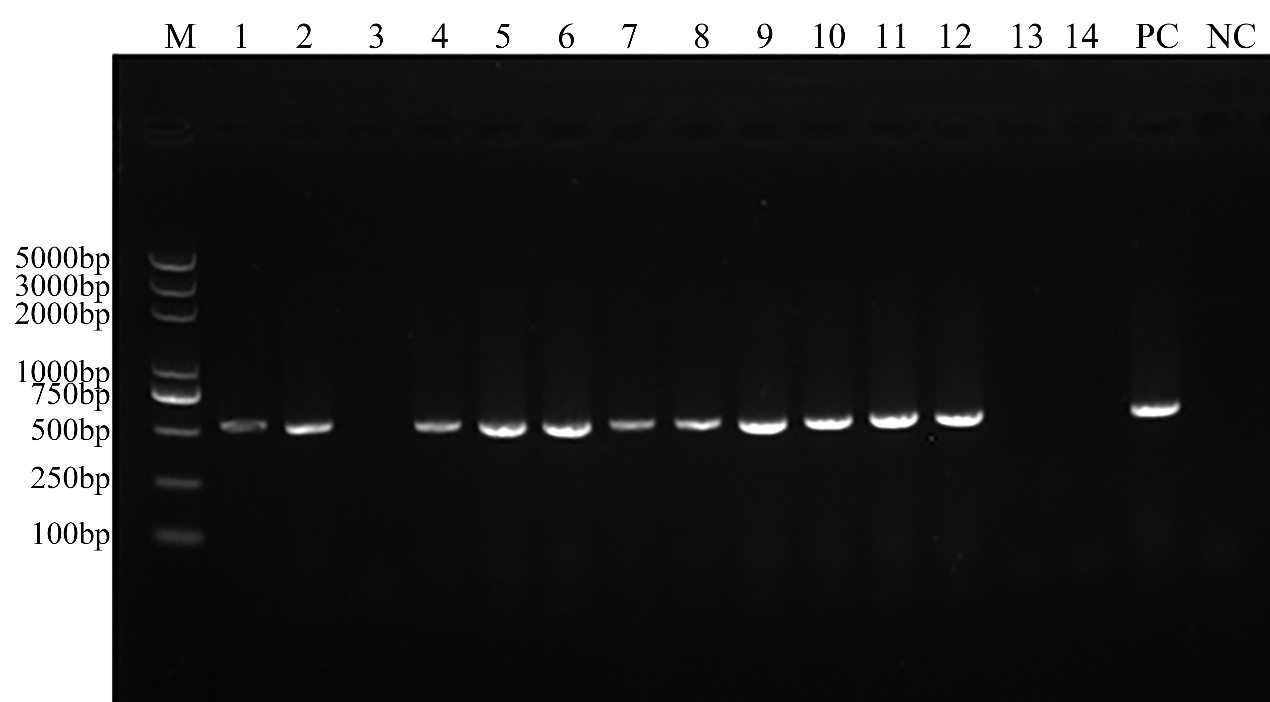


**Fig S7. Results of *T. gondii* nested PCR amplification based on the B1 locus.** 1-14: tissue samples; PC: Positive control; NC: Negative control.


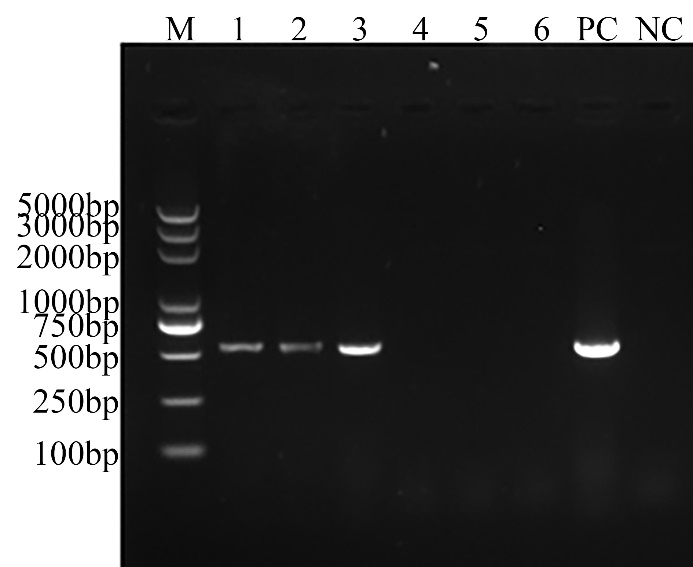


**Fig S8. Results of *T. gondii* nested PCR amplification based on the B1 locus.** 1-6: blood samples; PC: Positive control; NC: Negative control.

TABLE S3 Nucleotide sequences used in this study

|  | Nested PCR-Positive | Nested PCR-Negative | Total |
| --- | --- | --- | --- |
| REPORT-Positive | 11 tissue samples +  3 blood samples | 0 | 14 |
| REPORT-Negative | 0 | 3 tissue samples +  3 blood samples | 6 |
| Total | 14 | 6 | 20 |
